# OpenStreetMap-derived multimodal dataset across 23 cities: Paired urban morphology tiles with bioclimatic variables

**DOI:** 10.1016/j.dib.2026.112518

**Published:** 2026-01-27

**Authors:** Tao He, Wei Lu

**Affiliations:** School of Civil Engineering, Liaoning Technical University, Fuxin, Liaoning Province, China

**Keywords:** Machine learning, Geospatial AI, Geographic information system, Urban morphology, Urban planning, OpenStreetMap

## Abstract

We present an OpenStreetMap-derived multimodal dataset spanning 23 cities and 11,711 tile-level samples. For each 768 × 768 m tile, we provide an aligned image pair: (i) a stylized ecological baseline that generalizes green and water features together with major roads and railways, and (ii) a target urban morphology map color-coded by functional building classes, transport infrastructure, green space, and water. Each sample includes latitude/longitude; the eight WorldClim v2.1 bioclimatic variables can be reconstructed locally with the provided script. The dataset is organized by city and indexed with JSONL records linking image paths and attributes, enabling direct integration into machine learning pipelines. Cross-city and cross-climate coverage supports training and evaluation of generative models for urban design, comparative analyses of morphology across climate regimes, and imputation of functional footprints in data-scarce regions. The ecological baseline represents a constructed pre-urban template rather than a historical map.

Specifications Table

**Table:** 

Subject	Earth & Environmental Sciences
Specific subject area	Geospatial AI; Geographic Information Science; Urban morphology
Type of data	Image (PNG), Per-tile attributes (JSONL; coordinates only), Reproducibility script
Data collection	Vector data were acquired from OpenStreetMap (OSM) by extraction through OSMnx between March 2025 and July 2025. Data were collected on a city-level basis for 23 cities and were processed with GeoPandas and Shapely, then exported as Environmental Systems Research Institute (ESRI) Shapefile. Image tiles were batch-rendered at 768 × 768 pixels (1 m/pixel) using PyQGIS. Per-tile attributes include tile centroids. Bioclimatic variables from WorldClim v2.1 (30 arc-second resolution) can be reconstructed locally via the provided script; they are not redistributed in the public archive.
Data source location	Vector data of urban features were acquired from OpenStreetMap [1] (https://www.openstreetmap.org/) via OSMnx [2]. WorldClim [3] (https://www.worldclim.org/data/worldclim21.html) for bioclimatic variables. Ocean water polygons were obtained from the OSM Water Polygons dataset [4] (https://osmdata.openstreetmap.de/data/water-polygons.html).
Data accessibility	Repository name: Zenodo Data identification number: DOI: 10.5281/zenodo.17586383 Direct URL to data: https://doi.org/10.5281/zenodo.17586383
Related research article	None

## 1 Value of the Data

• This multimodal dataset comprises 11,711 samples, each aligned to a unique geographic tile. The primary modality is a paired set of raster images (PNG): (i) a simplified initial image (input; contextual base layer) and (ii) a target image (ground truth; semantic label map) color-coded by thematic classes (functional buildings, main roads, railways, green space, and water). Each sample has coordinates; the eight WorldClim bioclimatic variables can be reconstructed locally with the provided script.
• Researchers in GIScience, urban planning, and machine learning can use these data to (i) develop and explore attribute- or image-conditioned approaches for urban planning and design; (ii) analyze urban morphology across diverse climate regimes; and (iii) impute and complete functionally annotated building footprints and other vector layers in data-scarce regions. Cross-city and cross-climate coverage support comparative and transfer studies across cities and climate zones.
• All image pairs consist of 768 × 768 m tiles rendered at 768 × 768 pixels, using a consistent color legend with anti-aliasing disabled. The dataset is indexed by two global JSONL files: tiles.jsonl (train) and tiles_test.jsonl (test). Each line refers to a single sample stored in city-specific directories. This JSONL structure allows the data to be loaded directly into training and evaluation pipelines of common machine learning frameworks.

## 2 Background

Urban analysis and planning often rely on volunteer and open geospatial sources (e.g., OpenStreetMap), where building footprints, functional labels, and land-use attributes are uneven across cities. Public datasets for urban-form modelling are typically single-modality (e.g., roads-to-footprints) and emphasize geometry; recent generative/completion systems [5,6] seldom include functional semantics or environmental context. Moreover, urban climatology documents systematic links between morphology and the near-surface climate (e.g., local climate zones and bioclimatic design), which motivates the inclusion of climate covariates when studying urban forms and enables cross-city/cross-climate evaluation settings [[7], [8], [9]]. Against this background, we compiled a multimodal, globally distributed, tile-based dataset that pairs (i) an initial map (containing natural green and water features, core railway infrastructure and main roads), (ii) a target image (color-coded map of urban features), and (iii) per-tile location; bioclimatic variables can be reconstructed locally from WorldClim v2.1 [3].

## 3 Data Description

### 3.1 Dataset structure

**Fig. 1:**
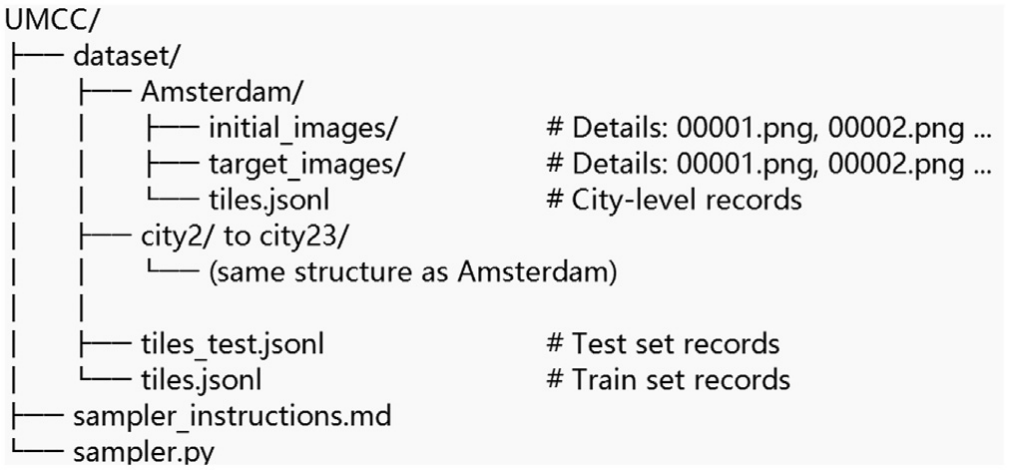
Root directory structure of the dataset.

The dataset is organized by city. The directory layout is shown in Fig. 1. Each city folder contains two subdirectories, initial_images and target_images, as well as a city-level tiles.jsonl file. In total, 23 city folders named by city (e.g., Amsterdam, Athens, etc.) are included, all of which follow the same structure. Additionally, at the dataset root, two global JSONL index files list all per-tile records: tiles.jsonl (train split) and tiles_test.jsonl (test split).

### 3.2 Image

**Fig. 2:**
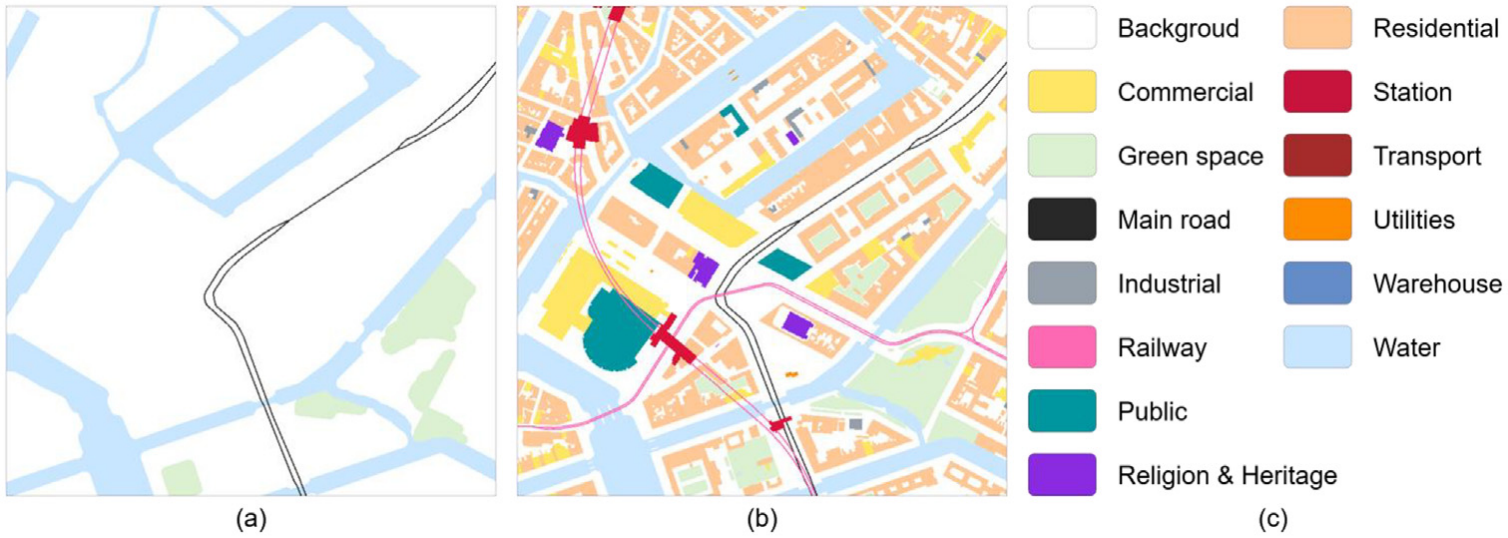
Sample image pairs. Each image patch is 768 × 768 pixels and was exported from QGIS with anti-aliasing disabled.

Fig. 2 shows the paired images: (a) A sample initial image representing the pre-development baseline, including green space, water, the arterial road network, and conventional (non-high-speed) railways. This layer does not represent an observed map; rather, it is an ecological baseline, constructed through spatial generalization of the target image’s green space and water features, intended to approximate pre-urbanization ecological conditions. (b) A sample target image depicting the existing urban morphology, including the city’s principal elements: building functions, the arterial road network, green space, water, and the railway network. (c) The encoding scheme used across all images, in which building elements are classified into eight functional categories: commercial, industrial, public, religious and heritage, residential, transport, utilities, and warehouse (Table 1).

**Table 1:** Color palette.

Class	RGB	Hex	Color
Residential	255, 200, 150	#ffc896	
Religious and Heritage	138, 43, 226	#8a2be2	
Public	0, 150, 160	#0096a0	
Commercial	255, 204, 0	#ffcc00	
Utilities	255, 140, 0	#ff8c00	
Industrial	150, 160, 170	#96a0aa	
Warehouse	100, 140, 200	#648cc8	
Transport	165, 42, 42	#a52a2a	
Railway	255, 105, 180	#ff69b4	
Station	220, 20, 60	#dc143c	
Main road	40, 40, 40	#282828	
Green space	220, 240, 210	#dcf0d2	
Water	200, 230, 255	#c8e6ff	

### 3.3 Data record structure

The dataset is provided in the JSON Lines (jsonl) format. Each line in the file represents a single data record as a self-contained JSON object. This format was chosen for its scalability and ease of use with modern data processing pipelines, as it allows for efficient streaming and parallel processing without loading the entire dataset into memory. Each record consists of the key-value pairs described in Table 2.

**Table 2:** Data record schema.

Key	Data Type	Description
target_image	String	The relative path to the target image file, resolved from the dataset's root directory.
initial_image	String	The relative path to the initial conditioning image file, resolved from the dataset's root directory.
attributes	float vector (public: 2; reconstructed: 10)	A float vector containing [lat, lon] in the public release; after local reconstruction, it contains [lat, lon, bio1, bio4, bio5, bio6, bio12, bio15, bio16, bio17]. See Section 3.1.

### 3.4 Per-tile attribute vector

Bioclimatic variables (3-10) are sampled from WorldClim v2.1 (30 arc-second resolution). The values are direct samples (float32) without additional scaling. In the public release, WorldClim-derived values are excluded. The repository provides a script (UMCC/sampler.py) to sample the eight variables locally after obtaining the raster files from the official source. Table 3 provides the data dictionary of the per‑tile attributes, listing the coordinates and the eight WorldClim covariates used in each record.

**Table 3:** Per-tile attribute vector schema.

Attribute	Description
lat	Latitude in WGS84 decimal degrees.
lon	Longitude in WGS84 decimal degrees.
bio1	Annual Mean Temperature.
bio4	Temperature Seasonality (standard deviation * 100).
bio5	Max Temperature of Warmest Month.
bio6	Min Temperature of Coldest Month.
bio12	Annual Precipitation.
bio15	Precipitation Seasonality (Coefficient of Variation).
bio16	Precipitation of Wettest Quarter.
bio17	Precipitation of Driest Quarter.

### 3.5 Example record

Below is a single-line example from a jsonl file.

Public release:

{``target_image'': ``./dataset/Amsterdam/target_images/00232.png'', ``initial_image'': ``./dataset/Amsterdam/initial_images/00232.png'', ``attributes'': [52.3685974, 4.9045683]}

After local reconstruction:

{``target_image'': ``./dataset/Amsterdam/target_images/00232.png'', ``initial_image'': ``./dataset/Amsterdam/initial_images/00232.png'', ``attributes'': [52.3685974, 4.9045683, 9.9541664, 532.098999, 21.8999996, 0.5, 874.0, 21.2416534, 278.0, 174.0]}

### 3.6 Dataset geographic distribution

**Fig. 3:**
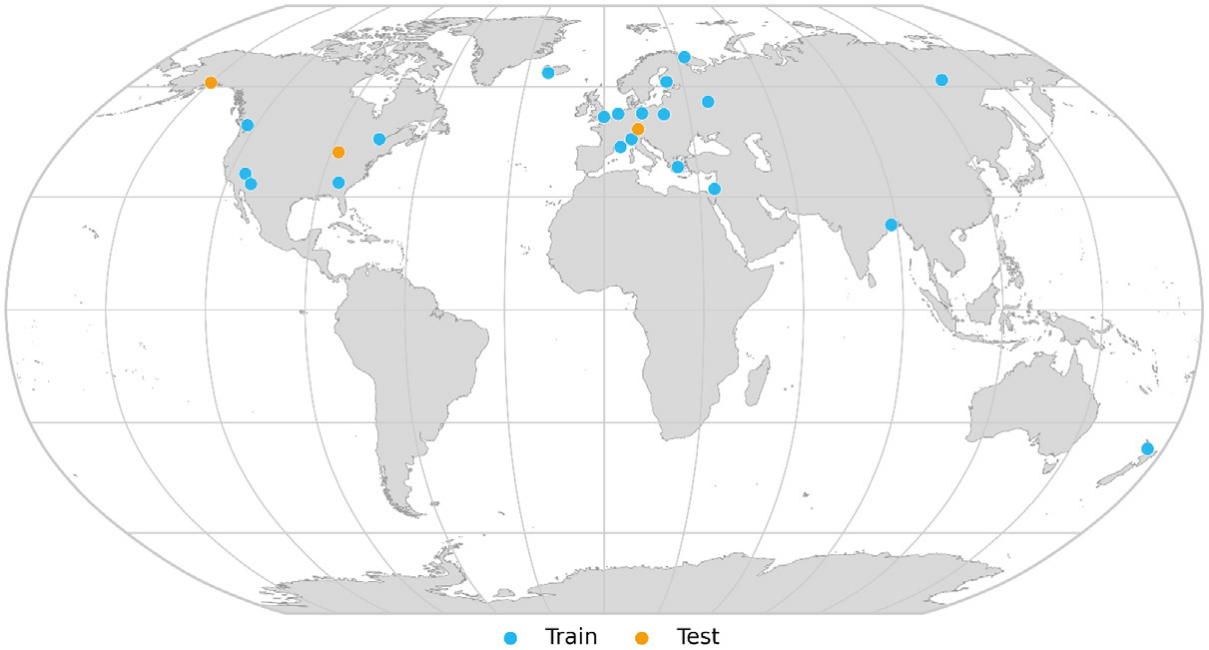
Geographic distribution of the 23 cities in the dataset. Training cities are shown in blue and testing cities in orange.

The dataset is partitioned into training and testing sets at the city level. Fig. 3 illustrates the geographic distribution of the 23 cities included in the dataset, indicating their assignment to either the training or testing set. A comprehensive table summarizing per-city summary information (including geographic coordinates, sampling bounding boxes, number of samples, and split assignment) is available as a CSV file (per_city_summary.csv) in the repository.

## 4 Experimental Design, Materials and Methods

**Fig. 4:**
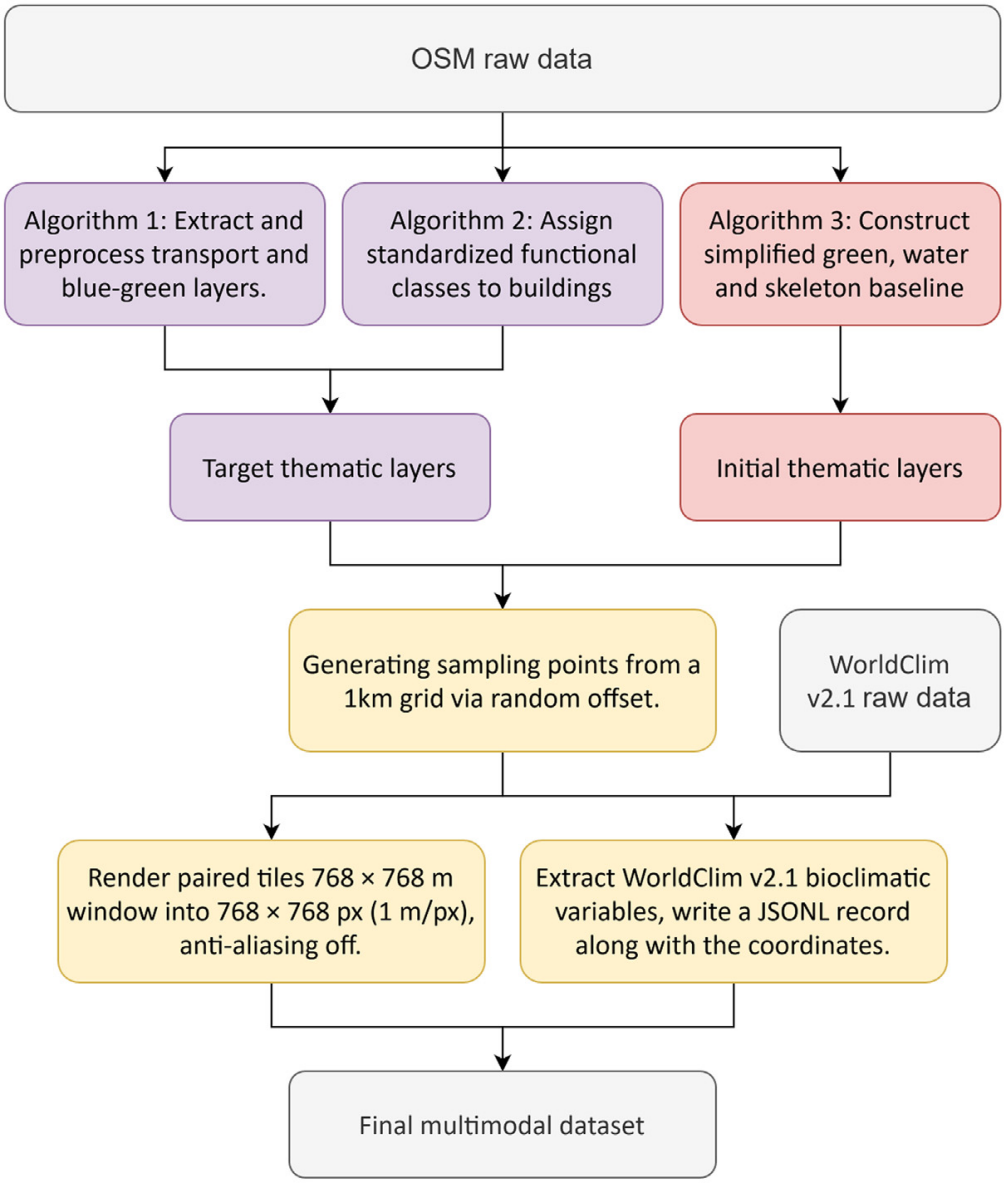
Overview of the data processing pipeline.

The generation of the multimodal dataset involves a multi-stage pipeline. The overall process, summarized in Fig. 4, consists of two main serial workflows: (i) vector data extraction and preprocessing to generate the target and initial thematic layers from OSM data, and (ii) patch sampling, image rendering, and covariates integration to produce the final paired dataset. The following subsections provide a detailed description of each stage.

### 4.1 Vector data extraction and preprocessing

For each city, we generated two distinct sets of ESRI Shapefile layers, termed initial and target, which serve as the inputs for image rendering. Each set is a collection of thematic layers (e.g., green space, water, and roads) that are stacked during rendering to create a composite map. The process was guided by predefined OpenStreetMap (OSM) tag filters (download_tags) curated for each layer in a central config.py file. These filters were designed to capture two distinct levels of macro urban morphology: the target layers utilize a comprehensive and fine-grained set of tags to represent the current urban fabric, whereas the initial layers employ a more conservative set to define a simplified baseline structure.

The general workflow for both layer sets involved programmatically extracting vector features using OSMnx via the Overpass API. The resulting data were uniformly reprojected to the local UTM zone (a metric CRS) and subsequently processed with a series of morphological operations (e.g., buffering, dissolving) in GeoPandas and Shapely. The final processed layers were then exported as ESRI Shapefiles. The specific workflows tailored for the target and initial layers are detailed in the following sections.

Vector processing environment: Python 3.9, OSMnx 2.0.0, GeoPandas 1.0.1, Shapely 2.0.6, Pyproj 3.6.1.

A configuration file (config.py) defines key parameters for all vector processing workflows. Specifically, it contains dictionaries that (i) specify the OpenStreetMap tag filters for each thematic layer (download_tags), and (ii) map building and land-use categories to standardized functional classes (building_to_class, landuse_to_class).

### 4.2 Target vector layers

The target layers aim to capture the detailed morphology and functional composition of the urban environment, encompassing residential (R), religious and heritage (RH), public (P), commercial (C), utilities (U), industrial (I), warehouse (W), and transport (T) areas. The generation process involves two main workflows, which handle building footprints separately from other features due to the unique requirement of functional label imputation.

First, thematic layers for green space, water, main roads, railways, and stations were extracted and processed. This workflow primarily involves downloading features based on predefined tags, reprojecting them to the local UTM coordinate system, and applying specific geometric rules (e.g., area filtering for green space). The main steps of this process are shown in Algorithm 1.

**Table:** Algorithm 1 Processing of target transport and blue–green layers.

**Step 1:** Download OSM features and reproject all geometries to the city’s local UTM coordinate system.
**Step 2:** Keep LineString/MultiLineString for roads and railways, and Polygon/MultiPolygon for green space, water, and stations.
**Step 3:** Remove small green-space polygons smaller than 25 m²
**Step 4:** Export the processed thematic layers (green space, water, railways, stations) and the shared main-roads layer as ESRI Shapefiles for rendering.

Second, a distinct workflow was required for the building layer to address the challenge of generic functional tags in OpenStreetMap (e.g., building=yes). The core of this workflow lies in assigning functions to generic buildings through a spatial overlay with the processed land-use layer and subsequently filtering out any unlabeled buildings. This strategy results in a building layer where every feature has a functional class. The full procedure is detailed in Algorithm 2.

**Table:** Algorithm 2 Processing and functional labeling of buildings.

**Step 1:** Download land-use and polygons then reproject all polygons to the city’s local UTM coordinate system.
**Step 2:** Remove overlaps between land-use features by applying small-polygon precedence to ensure mutual exclusivity.
**Step 3:** Download and reproject building polygons to the city’s local UTM coordinate system, then filter out small features (e.g., <25 m²).
**Step 4:** Label standardization map attributes to standardized classes. Imputes functional labels for generic buildings based on a spatial overlay with the processed land-use layer. For each building with a generic tag (e.g., building=yes), assigns the label from the overlapping land-use polygon with the largest intersection area, then drop unlabeled features.
**Step 5:** Export the labeled building layer in ESRI Shapefile format.

### 4.3 Initial vector layers

Unlike the target layers, the initial layers were constructed to represent a simplified ecological baseline and spatial skeleton of each city rather than an observed or historical map. We restricted the OSM tag set during extraction. For example, the target green space layer includes parks (leisure=park), whereas the initial layer keeps only natural features such as forests (natural=wood). Similarly, the railway and stations layers were restricted to primary rail infrastructure (railway=rail, railway=station), omitting secondary systems such as tram or subway networks that are retained in the target composition. This filtering abstracts the ecological and infrastructural skeleton, providing a stylized pre-urban baseline. The complete workflow is summarized in Algorithm 3.

**Table:** Algorithm 3 Construction of the simplified ecological/skeleton baseline.

**Step 1:** Download OSM features then reproject all geometries to the city’s local UTM coordinate system.
**Step 2:** For green space, connect nearby polygons (gap < 4 m), remove small patches (< 500 m²), and apply a net 8 m generalization (buffer–shrink).
**Step 3:** For water, connect features (gap < 20 m) and remove small patches (< 1500 m²) without net expansion or boundary smoothing.
**Step 4:** Filter stations by proximity to railways (50 m buffer) and export all initial layers as ESRI Shapefiles for rendering.

The specific vector processing steps outlined in Algorithm 1, 2 and 3, such as filtering, remove overlaps, spatial join, and geometric refinement, are implemented using a set of modular functions (OP1-OP6) defined in the provided download_core_function.py script.

### 4.4 Quality control

The initial green space and water ESRI shapefile are imported into QGIS and cleaned via delete holes (QGIS, native:deleteholes) with a minimum hole-area threshold (m²) to remove small interior holes.

For coastal cities, an additional step was performed to ensure a complete representation of water bodies. A sea polygon from the OSM Water Polygons dataset [4] was manually imported, reprojected to the city’s local UTM coordinate system, and clipped to the study area. It was then added as a separate layer in both the initial and target map compositions. For rendering, the sea layer used the same symbology as the water layer and was placed directly below the corresponding water layer in the stack. In this work, water collectively refers to inland water features and, where applicable, the sea polygon.

### 4.5 Patch sampling, rendering, and attribute integration

This section describes the per-city workflow used to generate the paired-image dataset. We first sampled patch centers within each city boundary. For each center, we rendered a spatially aligned image pair—the ecological and skeleton baseline map (the initial state) and the target urban morphology map (the target state)—using a consistent projection, scale, and resolution. Finally, we extracted the WorldClim bioclimatic variables at each center and wrote them, together with the corresponding coordinates, to the JSONL index of per-tile attributes.

Rendering environment: QGIS 3.16.0 Python interpreter (Python 3.7).

### 4.6 Point selection strategy

We constructed a regular 1 km grid within each city boundary. Around every grid node, we generated three candidate centers by adding a small random jitter, with independent offsets in x and y drawn uniformly from −400 to 400 m. For each candidate, if any bioclimatic variable was missing, the point was discarded and replaced by a randomly resampled point within the city boundary (up to 20 retries) to maintain the intended sample size. The implementation details are provided in get_center_list.py (generate_grid_points(), generate_patches(), and try_valid_point()).

### 4.7 Rendering strategy

For each center, we rendered an image pair under a common UTM projection and scale. Each tile covered a 768 m × 768 m square window centered at the point and was exported at 768 × 768 pixels (1 m/pixel) with anti-aliasing disabled. The layer stack was explicitly ordered as follows. Initial image layout (from bottom to top): green space, water, main roads, railways, and stations. Target image layout (from bottom to top): green space, water, main roads, buildings, railways, stations. Polygons were drawn as solid fills without outlines, and line layers used single-symbol styles with anti-aliasing disabled.

### 4.8 Attributes integration

At each patch center, we extracted eight WorldClim bioclimatic variables (bio1, bio4, bio5, bio6, bio12, bio15, bio16, bio17) and then wrote a single JSON record per sample to the city-level tiles.jsonl. Each record contains target_image and initial_image (relative paths from the dataset root) and attributes, a 10-float array ordered [lat, lon, bio1, bio4, bio5, bio6, bio12, bio15, bio16, bio17] sampled from WorldClim v2.1 (30 arc-second resolution) and stored as float32 without additional scaling. In the public release, we do not distribute WorldClim-derived values. To reproduce the full 10-float vector, users must first download the WorldClim v2.1 rasters from the official website and then run the provided script (UMCC/sampler.py), which reads the locally stored rasters and samples the variables at each tile center.

### 4.9 Quality control

Because only labeled building features from building layer were rendered, we inspected each target image alongside the corresponding map window. Samples were discarded if the share of non-background pixels was too low or if many unlabeled buildings were present, indicating missing features.

## Limitations

The initial (pre-development) image is a stylized ecological baseline constructed by generalizing green and water layers rather than a historical snapshot. When used as a conditioning input to predict the target, it may convey a coarse spatial structure correlated with the target. Functional labels for buildings inferred from land-use polygons can mislabel generic buildings where OSM tags are sparse or inconsistent.

## Ethics Statement

The proposed data do not involve human subjects, animal experiments, or data collected from social media platforms.

## Credit Author Statement

**Tao He:** Conceptualization, Methodology, Software, Data curation, Writing, Original draft preparation; **Wei Lu:** Conceptualization, Methodology, Supervision, Writing-Reviewing and Editing.

## Data Availability

ZenodoUMCC Dataset: Paired Urban Morphology Tiles with Per-Tile Coordinates and WorldClim Bioclimatic Variables (Original data).
